# Evaluation of Predictive and Prognostic Importance of Lung Immune Prognostic Index in Locally Advanced Rectal Cancer Patients Treated With Neoadjuvant Chemoradiotherapy

**DOI:** 10.7759/cureus.40548

**Published:** 2023-06-17

**Authors:** Rukiye Arıkan, Hilal Alkış, Selver Işık, Alper Yaşar, Abdussamet Çelebi, Nargiz Majidova, Nadiye Sever, Mustafa Adlı, Nazım C Demircan

**Affiliations:** 1 Department of Medical Oncology, Marmara University School of Medicine, Istanbul, TUR; 2 Department of Radiation Oncology, Marmara University School of Medicine, Istanbul, TUR; 3 Department of Medical Oncology, Erzurum Education and Training Hospital, Erzurum, TUR

**Keywords:** rectal cancer, prognosis, pathological response, neoadjuvant chemoradiotherapy, lung immune prognostic index

## Abstract

Objective: The systemic inflammatory response (SIR) is known as an important factor associated with tumorigenesis and tumor progression, and can be reflected by inflammatory markers. One of the markers that reflect this is the lung immune prognostic index (LIPI). It is based on a derived neutrophil-to-lymphocyte ratio (dNLR) and lactate dehydrogenase (LDH) level. We aimed to investigate the significance of LIPI in locally advanced rectal cancer (LARC) patients treated with neoadjuvant chemoradiotherapy (NACRT).

Methods: In this retrospective study, we stratified the patients according to LIPI score as good LIPI and intermediate (int)/poor LIPI. According to pathological response to NACRT, we divided the patients into two groups as those with complete response (CR) or near-CR, and those with partial response (PR) or poor/no response. We classified CR and near-CR as good response. We evaluated the predictive and prognostic significance of LIPI for NACRT response, disease-free survival (DFS), and overall survival (OS) by univariate and multivariate analyses.

Results: We included 137 patients in the results, with 72 (52.6%) having good LIPI and 65 (47.4%) having int/poor LIPI. The median follow-up period was 44.7 months (range: 10-105 months). Thirteen patients (18.0%) in the good LIPI group and 22 patients (34.0%) in the int/poor LIPI group achieved good response. In multivariate analysis, we found only the LIPI score as an independent risk factor (hazard ratio (HR): 2.4, p = 0.04) for NACRT response. Median DFS was 89.2 months (95% CI: 11.4-167.0) in the int/poor LIPI group; however, the DFS of all study populations and patients in the good LIPI group did not reach the median value. In multivariate analysis for DFS, we identified abdominoperineal resection (APR) (HR: 2.21, p = 0.02), presence of tumor deposit (HR: 2.96, p = 0.003), and int/poor LIPI score (HR: 2.07, p = 0.02) as separate risk variables. OS of all study populations and the patients in the LIPI groups did not reach the median value. In multivariate analysis for OS, we identified APR (HR: 2.74, p = 0.02), surgical margin positivity (HR: 12.94, p < 0.001), and adjuvant CT (HR: 0.20, p = 0.002) as separate risk variables for OS.

Conclusion: This is the first study investigating the predictive and prognostic significance of LIPI in LARC patients treated with NACRT. The results revealed that int/poor LIPI was associated with a higher rate of good response but shorter DFS compared to good LIPI. The baseline LIPI score serves as an easily accessible and useful prognostic index, and it has significant potential for making appropriate treatment decisions in LARC.

## Introduction

Rectal cancer (RC) is the eighth most common type of cancer worldwide and accounts for approximately 40% of all colorectal malignancies [1]. The standard treatment for locally advanced rectal cancer (LARC) is total neoadjuvant therapy, which includes neoadjuvant chemoradiotherapy (NACRT) plus chemotherapy (CT) or vice versa, followed by total mesorectal excision (TME) [2]. However, until recently, NACRT followed by TME and adjuvant CT have been considered as standard treatment strategy [3-5]. NACRT offers a higher probability of tumor downstaging and better local and systemic control [6,7]. However, patients receiving NACRT have a wide variation in oncological outcomes that are affected by many variables. Pathologic response to NACRT is the most reported variable affecting survival outcomes and it significantly differs among RC patients [8,9]. It is important to identify predictive factors for treatment response and survival outcomes, for accurate patient selection and monitoring.

The systemic inflammatory response (SIR) has been a widely emphasized factor associated with cancer development, progression, and metastasis. The association of various biomarkers, such as neutrophil-to-lymphocyte ratio (NLR), platelet-to-lymphocyte ratio (PLR), monocyte-to-lymphocyte ratio (MLR), modified Glasgow prognostic score (mGPS), and systemic immune-inflammation index (SII), with SIR and oncological outcomes has been studied in several cancer types, including LARC [10-12]. A number of studies have also demonstrated a relationship between SIR and radiotherapy (RT) response [13,14].

An innovative index called the lung immune prognostic index (LIPI) was researched in patients treated with immune checkpoint inhibitors (ICIs) for different cancer types [15-17]. LIPI was first studied by Mezquita et al. in non-small cell lung cancer patients treated with ICIs and found to be associated with SIR and prognosis [18]. LIPI is calculated using two clinical biomarkers, which are both widely used in daily clinical practice. It is based on a derived NLR (dNLR) ≥ 3 and a pretreatment lactate dehydrogenase (LDH) level ≥ the upper limit of normal (ULN). Thus, it serves as an easily accessible and useful index in the prediction of prognosis. As far as the literature available, the predictive value of LIPI for treatment response and survival outcomes has never been investigated in LARC patients treated with chemoradiotherapy (CRT).

The present study sought to investigate the predictive and prognostic value of LIPI in LARC patients treated with NACRT.

## Materials and methods

Study population and data collection

We included LARC patients in this study and followed them up from 2012 to 2021 in our center. Inclusion criteria were as follows: (1) patients with histologically proven RC; (2) who had stage II or III disease according to the American Joint Committee on Cancer's (AJCC) tumor, node, and metastasis (TNM) staging system; (3) who received a long course NACRT; and (4) who underwent TME. We retrospectively reviewed data, including baseline characteristics of the patients, clinical tumor staging, ypTNM stage and other pathological findings, history of adjuvant CT, and laboratory data. Pathological findings also included histology, number of resected lymph nodes, lymphovascular invasion (LVI), perineural invasion (PNI), presence of tumor deposit, and surgical margin. Tumor deposits were extramural discontinuous cancer spreads located in the subserosa, mesentery, non-peritonealized pericolic, or perirectal/mesorectal tissues. We evaluated clinical tumor staging by colonoscopy, chest computed tomography, abdominopelvic magnetic resonance image (MRI), or computed tomography. The Local Ethics Committee of our center approved the research protocol (approval number: 09.2021.1030).

Definition of LIPI score

We calculated the LIPI scores according to dNLR (absolute neutrophil count/(white blood cell count − absolute neutrophil count)) and LDH levels. According to the LIPI score, we divided the patients into three groups as follows: good (dNLR < 3 and LDH normal), intermediate (int) (dNLR ≥ 3 or LDH ≥ ULN), and poor (dNLR ≥ 3 and LDH ≥ ULN), as reported by Mezquita et al. [18].

Treatment and follow up

Radiation oncologists of our center applied volumetric modulated arc therapy (VMAT) using RapidArc delivery of 6 MV X-Rays (Eclipse TPS, Clinac, Varian Medical Systems, Palo Alto, CA). They prescribed a dose of 50.4 to 56 Gy for the planning target volume (PTV) of the tumor and 45 to 50.4 Gy for the PTV of lymph nodes. They applied all target volumes simultaneously in 25 to 28 fractions of 1.8 to 2 Gy. PTV coverage was at least 95% of the prescribed dose. They evaluated treatment fields with daily kV imaging. All patients received capecitabine (825 mg/m2 orally twice daily for five days/week during CRT) concurrently with RT. Colorectal surgeons performed TME within a median of eight to 12 weeks after completion of NACRT. The multidisciplinary tumor board decided on adjuvant CT by evaluating the performance status and clinical and pathological stages of the patients. We followed up with the patients every three months in the first two years after surgery and every six months afterward. We evaluated chest computed tomography and abdominal computed tomography or MRI every six months in the first three years and then once a year. Patients had total colonoscopy one year after surgery and every two years thereafter.

Response assessment and survival outcomes

We used to pathological tumor regression grade (TRG) system [19] to evaluate the pathological tumor response to NACRT. Disease-free survival (DFS) is defined as the time between curative surgery and local or distant recurrence, death, or the last medical examination. Overall survival (OS) is defined as the time between curative surgery and death or the last medical examination.

Statistical analysis

We stratified the study cohort into two groups as patients with good LIPI scores and patients with int/poor LIPI scores. According to pathological response to NACRT, we divided the patients into two groups as those with complete response (CR) or near-CR, and those with partial response (PR) or poor/no response. We classified CR and near-CR as good response. We evaluated LDH, carcinoembryonic antigen (CEA), and cancer antigen (CA) 19-9 by using the cut-off value of our laboratory (240 U/L, 5 μg/L, and 34 U/mL, respectively). We used frequencies and percentages to represent descriptive data, interquartile ranges (IQRs) to illustrate the median values for continuous variables, the chi-square test to compare categorical variables, and the Kaplan-Meier method and the log-rank test to estimate survival. We used the Cox proportional and logistic regression models to detect variables significantly affecting the outcomes or those tending toward significance (p < 0.10) in univariate analyses. We used the backward-stepwise method in multivariate analyses. We accepted 95% and 0.05 for the confidence interval (CI) and p-value, respectively.

## Results

The study included 137 patients (86 males and 51 females) with a median age of 58 years (IQR, 50-65). Table 1 summarizes the baseline characteristics, histopathological features, and treatments. Seventy-two patients (52.6%) had a good LIPI score, and 65 patients (47.4%) had an int/poor LIPI score. The median interval between TME and completion of NACRT was 10.3 months (IQR: 7.9-11.8).

**Table 1:** Demographic and clinical characteristics of the study subjects CEA: cancer embryonic antigen; CA: cancer antigen; CT: chemotherapy; IQR: interquartile range; LIPI: lung immune prognostic index; LVI: lymphovascular invasion; PNI: perineural invasion; TRG: tumor regression grade. "+" refers to the existence of variable and "-" refers to the absence of variable.

	All	Good LIPI	Int/Poor LIPI	P-value
	n = 137	n = 72	n = 65	
Age, years				
Median (IQR)	58 (50-65)	57 (51-64)	60 (49-67)	0.55
Gender, n (%)				
Female	51 (37.2)	27 (37.5)	24 (36.9)	0.94
Male	86 (62.8)	45 (62.5)	41 (63.1)	
Histology				
Pure adenocarcinoma	124 (90.5)	65 (90.3)	59 (90.8)	0.92
Mucinous	6 (4.3)	4 (5.5)	2 (3.1)	
Signet ring cell	7 (5.1)	3 (4.2)	4 (6.1)	
Clinical stage, n (%)				
Stage II	15 (11.0)	9 (12.5)	6 (9.2)	0.54
Stage III	122 (89.0)	63 (87.5)	59 (90.8)	
cT, n (%)				
2	42 (30.7)	28 (38.9)	14 (21.5)	0.07
3	78 (56.9)	35 (48.6)	43 (66.2)	
4	17 (12.4)	9 (12.5)	8 (12.3)	
cN, n (%)				
0	15 (10.9)	9 (12.5)	6 (9.2)	0.46
1	98 (71.5)	53 (73.6)	45 (69.2)	
2	24 (17.5)	10 (13.9)	14 (21.5)	
Distance from the anal verge, n (%)				
0-6 cm	73 (53.2)	41 (56.9)	32 (49.2)	0.66
7-11 cm	52 (38.0)	25 (34.7)	27 (41.5)	
≥12 cm	12 (8.8)	6 (8.3)	6 (9.2)	
CEA, n (%)				
<5 (μg/L)	74 (61.6)	43 (67.2)	31 (55.4)	0.18
≥5 (μg/L)	46 (38.4)	21 (32.8)	25 (44.6)	
CA 19-9, n (%)				
<34 (U/mL)	82 (67.5)	49 (86.0)	33 (82.5)	0.64
≥34 (U/mL)	15 (12.5)	8 (14.0)	7 (17.5)	
Surgical technique				
Anterior resection	96 (70.1)	45 (62.5)	51 (78.5)	0.04
Abdominoperineal resection	41 (29.9)	27 (37.5)	14 (21.5)	
Number of resected lymph nodes, n (%)				
<12	73 (53.3)	42 (58.3)	31 (47.7)	0.21
≥12	64 (46.7)	30 (41.7)	34 (52.3)	
Tumor deposits, n (%)				
+	25 (18.2)	10 (13.9)	15 (23.1)	0.16
-	112 (81.8)	62 (86.1)	50 (76.9)	
LVI, n (%)				
+	41 (30.0)	23 (31.9)	18 (27.7)	0.59
-	96 (70.0)	49 (68.1)	47 (72.3)	
PNI, n (%)				
+	25 (18.2)	13 (18.1)	12 (18.5)	0.95
-	112 (81.8)	59 (81.9)	53 (81.5)	
Surgical margin				
+	6 (4.4)	3 (4.2)	3 (4.6)	0.89
-	131 (95.6)	69 (95.8)	62 (95.4)	
TRG, n (%)				
Score 0+1	35 (25.6)	13 (18.1)	22 (33.8)	0.03
Score 2+3	102 (74.4)	59 (81.9)	43 (66.2)	
Adjuvant CT, n (%)				
+	118 (86.1)	63 (87.5)	55 (84.6)	0.63
-	19 (13.9)	9 (12.5)	10 (15.4)	

In the entire cohort, NACRT responses according to TRG score were as follows: CR (TRG score: 0) in 22 patients (16.1%), near-CR (TRG score: 1) in 13 patients (9.5%), PR (TRG score: 2) in 71 patients (51.8%), and poor/no response (TRG score: 3) in 31 patients (22.6%). Thirteen patients (18.0%) in the good LIPI group and 22 patients (34.0%) in the int/poor LIPI group achieved good response. Table 2 shows the response assessment in the LIPI groups and all study populations. In univariate analyses, poor Eastern Cooperative Oncology Group Performance Status (ECOG-PS) and int/poor LIPI score were associated with good response (p = 0.04, for both). In multivariate analysis, only the LIPI score was an independent risk factor in treatment response. The int/poor LIPI score increased the probability of achieving a good response by 2.4 times (95% CI: 1.06-5.51) compared to a good LIPI score. Table 3 presents univariate and multivariate analyses for NACRT response.

**Table 2:** Response assessment and survival outcomes CR: complete response; CI: confidence interval; DFS: disease-free survival; Int: intermediate; HR: hazard ratio; LIPI: lung immune prognostic index; NACRT: neoadjuvant chemoradiotherapy; NR: not reached; OR: odds ratio; OS: overall survival; PR: partial response.

	Good LIPI	Int/poor LIPI	All study population
	n = 72	n = 65	n = 137
NACRT response, n (%)			
CR + near-CR	13 (18.0)	22 (34.0)	35 (25.6)
PR + poor/no response	59 (82.0)	43 (66.0)	102 (74.4)
OR (95% CI)	2.32 (1.05-5.12)		
p	0.04		
DFS			
Events, n (%)	16 (22.2)	23 (35.4)	39 (28.5)
Median DFS, months	NR	89.2	NR
HR (95% CI)	1.91 (1.01-3.61)		
p	0.04		
OS			
Events, n (%)	11 (15.3)	14 (21.5)	25 (18.2)
Median OS, months	NR	NR	NR
HR (95% CI)	1.57 (0.71-3.47)		
p	0.26		

**Table 3:** Univariate and multivariate analyses for NACRT response CEA: cancer embryonic antigen; CA: cancer antigen; CI: confidence interval; CT: chemotherapy; ECOG-PS: Eastern Cooperative Oncology Group-Performance Status; Int: intermediate; LIPI: lung immune prognostic index; NACRT: neoadjuvant chemoradiotherapy; OR: odds ratio; pCR: pathological complete response; Ref: reference. "+" refers to the existence of variable and "+" refers to the absence of variable.

	pCR/near pCR (%)	Univariate analyses		Multivariate analysis	
		OR (95% CI)	p-value	OR (95% CI)	p-value
Age, years					
<58	22	Ref.	0.36		
≥58	29	1.44 (0.66-3.15)			
Gender					
Female	20	Ref.	0.05	Ref.	0.08
Male	35	2.21 (1.01-4.84)		2.10 (0.92-4.79)	
ECOG-PS					
0	21	Ref.	0.04	Ref.	0.06
1 or 2	40	2.43 (1.03-5.78)		2.43 (0.98-6.02)	
CEA					
<5 (μg/L)	29	1.64 (0.70-3.88)	0.26		
≥5 (μg/L)	20	Ref.			
CA 19-9					
<34 (U/mL)	27	2.41 (0.52-11.26)	0.26		
≥34 (U/mL)	13	Ref.			
Histology					
Pure adenocarcinoma	23	Ref.	0.08	Ref.	0.13
Mucinous + signet ring cell	46	2.81 (0.87-9.02)		2.65 (0.76-9.22)	
Clinical stage					
Stage II	33	1.53 (0.49-4.84)	0.47		
Stage III	25	Ref.			
LIPI score					
Good	18	Ref.	0.04	Ref.	0.04
Int/poor	34	2.32 (1.05-5.12)		2.41 (1.06-5.51)	

**Figure 1:**
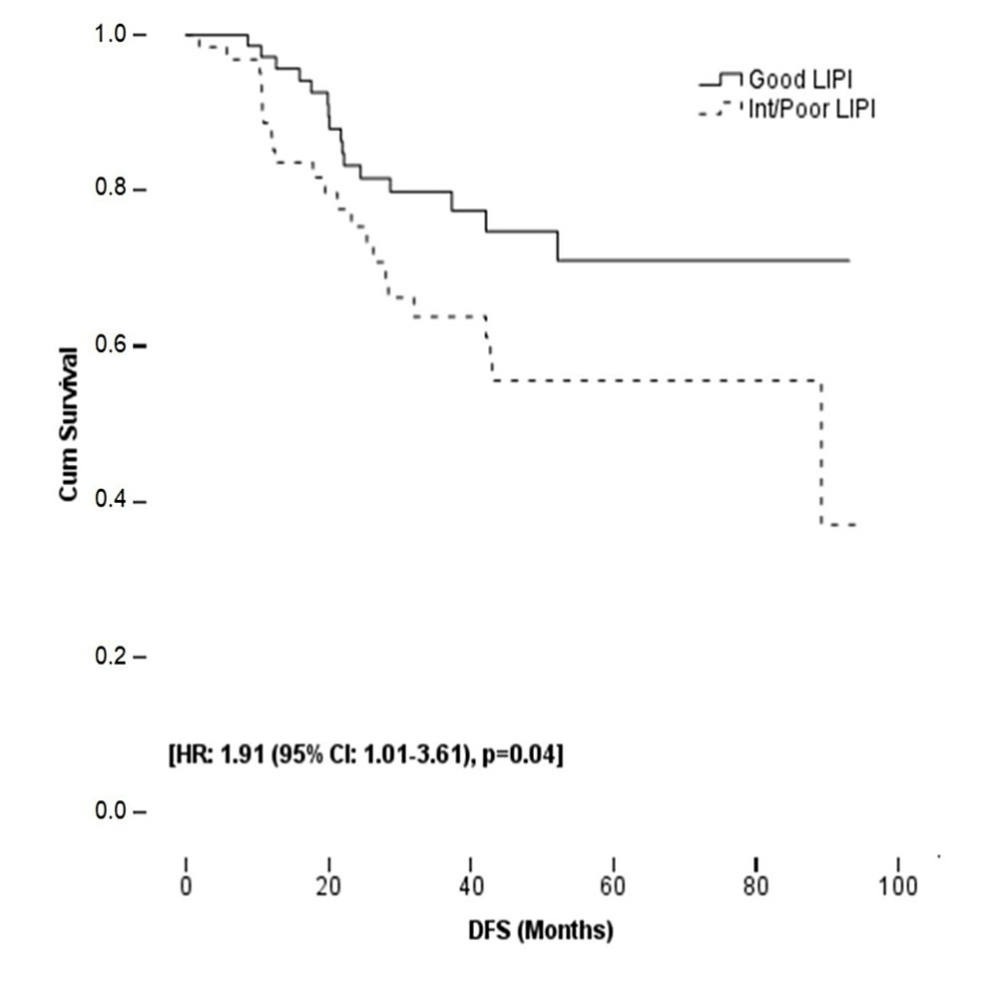
The Kaplan-Meier curve for DFS according to LIPI groups DFS: disease-free survival; LIPI: lung immune prognostic index.

The median follow-up period was 44.7 months (range: 10-105 months). During the follow-up period, 39 (28.5%) patients developed recurrence, seven were local recurrence and 32 were distant metastasis. Median DFS was 89.2 months (95% CI: 11.4-167.0) in the int/poor LIPI group; however, the DFS of all study populations and patients in the good LIPI group did not reach the median value. Table 2 shows survival outcomes in the LIPI groups and all study populations. In univariate analyses, the presence of tumor deposit, PNI, and int/poor LIPI score was associated with DFS (p = 0.004, p = 0.01, and p = 0.04, respectively). In multivariate analysis, abdominoperineal resection (APR), presence of tumor deposit, and int/poor LIPI score were independent poor prognostic factors for DFS (p = 0.02, p = 0.003, and p = 0.02, respectively). The risk of recurrence was 2.07 times higher in the int/poor LIPI group (95% CI: 1.08-3.98) than in the good LIPI group. Although there was a numerical difference in DFS between the patients who received and who did not receive adjuvant CT, it did not reach statistical significance. While the DFS of patients with negative surgical margins did not reach the median value, it was 28.3 months in patients with positive surgical margins. However, in multivariate analysis, there was no significant relationship between surgical margin and DFS. Figure 1 and Table 4 show the Kaplan-Meier curve and univariate and multivariate analyses for DFS, respectively.

**Table 4:** Univariate and multivariate analyses for disease-free survival CEA: cancer embryonic antigen; CA: cancer antigen; CI: confidence interval; CT: chemotherapy; DFS: disease-free survival; HR: hazard ratio; Int: intermediate; IQR: interquartile range; LIPI: lung immune prognostic index; LVI: lymphovascular invasion; mDFS: median disease-free survival; NR: not reached; PNI: perineural invasion; Ref: reference; TRG: tumor regression grade. "+" refers to the existence of variable and "+" refers to the absence of variable.

	Univariate analyses			Multivariate analysis	
	mDFS (months) (95% CI)	HR (95% CI)	p	HR (95% CI)	p
Age, years					
<58	NR	Ref.	0.20		
≥58	89.2 (NR)	0.66 (0.35-1.25)			
Gender					
Female	NR	1.29 (0.68-2.43)	0.43		
Male	NR	Ref.			
Histology					
Pure adenocarcinoma	NR	Ref.	0.81		
Mucinous + signet ring cell	NR	1.13 (0.40-3.20)			
Clinical stage					
Stage II	NR	Ref.	0.22		
Stage III	89.2 (NR)	2.05 (0.63-6.69)			
Distance from the anal verge					
0-6 cm	NR	Ref.	0.75		
7-11 cm	89.2 (NR)	0.88 (0.45-1.72)			
≥12 cm	NR	0.60 (0.14-2.53)			
CEA					
<5 (μg/L)	NR	Ref.	0.80		
≥5 (μg/L)	NR	1.10 (0.54-2.22)			
CA 19-9					
<34 (U/mL)	NR	Ref.	0.94		
≥34 (U/mL)	NR	1.05 (0.36-3.06)			
Surgical technique					
Anterior resection	NR	Ref.	0.09	Ref.	0.02
Abdominoperineal resection	NR	1.71 (0.90-3.26)		2.21 (1.13-4.31)	
Number of resected lymph nodes					
<12	NR	Ref.	0.14		
≥12	89.2 (NR)	0.62 (0.32-1.18)			
Tumor deposits					
+	28.4 (13.0-43.8)	2.76 (1.36-5.62)	0.004	2.96 (1.43-6.13)	0.003
-	NR	Ref.		Ref.	
LVI					
+	NR	1.53 (0.80-2.92)	0.19		
-	89.2 (NR)	Ref.			
PNI					
+	37.3 (19.2-55.3)	2.31 (1.16-4.58)	0.01	1.72 (0.78-3.78)	0.18
-	NR	Ref.		Ref.	
Surgical margin					
+	28.3 (15.3-41.5)	2.77 (0.84-9.13)	0.08	1.19 (0.33-4.31)	0.79
-	NR	Ref.		Ref.	
TRG					
Score 0+1	NR	0.59 (0.26-1.34)	0.20		
Score 2+3	89.2 (NR)	Ref.			
LIPI score					
Good	NR	Ref.	0.04	Ref.	0.02
Int/poor	89.2 (11.4-167.0)	1.91 (1.01-3.61)		2.07 (1.08-3.98)	
Adjuvant CT					
+	89.2 (NR)	0.61 (0.27-1.37)	0.22		
-	42.1 (NR)	Ref.			

**Figure 2:**
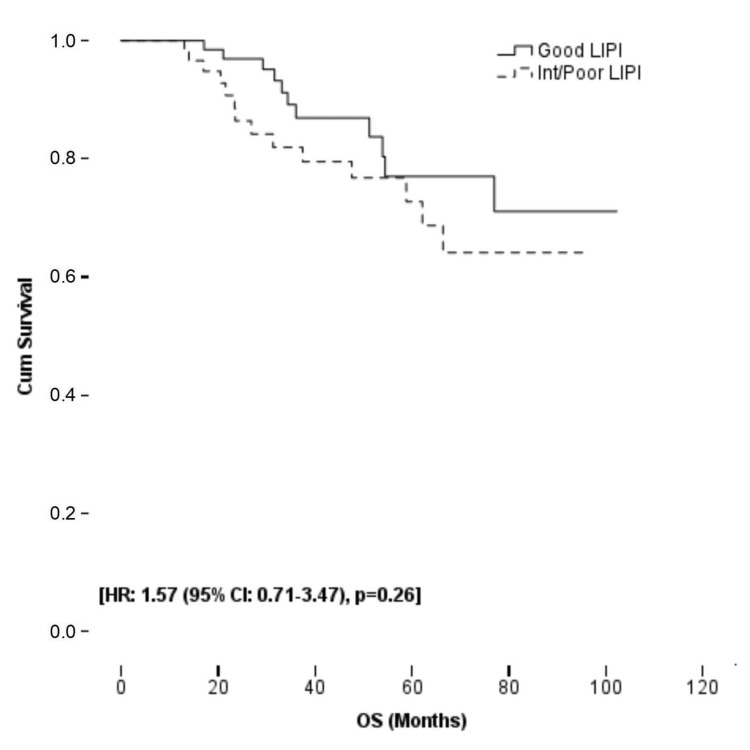
The Kaplan-Meier curve for OS according to LIPI groups LIPI: lung immune prognostic index; OS: overall survival.

Twenty-five patients (18.2%) died during the follow-up period. OS of all study populations and patients in the LIPI groups did not reach the median value. In univariate analyses, surgical margin positivity and absence of adjuvant CT were associated with OS (p < 0.001 and p = 0.02, respectively). In multivariate analysis, APR, surgical margin positivity, and absence of adjuvant CT were independent prognostic factors for OS (p = 0.02, p < 0.001, and p = 0.002, respectively). The LIPI score was not associated with OS. While surgical margin positivity worsened OS by 12.9 (95% CI: 3.9-42.8) times, adjuvant CT reduced the risk of death by 80% (95% CI: 0.07-0.55). Figure 2 and Table 5 show the Kaplan-Meier curve and univariate and multivariate analyses for OS, respectively.

**Table 5:** Univariate and multivariate analyses for overall survival CEA: cancer embryonic antigen; CA: cancer antigen; CI: confidence interval; CT: chemotherapy; HR: hazard ratio; Int: intermediate; IQR: interquartile range; LIPI: lung immune prognostic index; LVI: lymphovascular invasion; mOS: median overall survival; NR: not reached; OS: overall survival; PNI: perineural invasion; Ref: reference; TRG: tumor regression grade. "+" refers to the existence of variable and "-" refers to the absence of variable.

	Univariate analyses			Multivariate analysis	
	mOS (months) (95% CI)	HR (95% CI)	p	HR (95% CI)	p
Age, years					
<58	NR	Ref.	0.29		
≥58	NR	1.53 (0.69-3.42)			
Gender					
Female	NR	0.78 (0.34-1.81)	0.56		
Male	NR	Ref.			
Histology					
Pure adenocarcinoma	NR	Ref.	0.58		
Mucinous + signet ring cell	NR	1.65 (0.56-4.84)			
Clinical stage					
Stage II	NR	Ref.	0.35		
Stage III	NR	1.96 (0.46-8.35)			
Distance from the anal verge					
0-6 cm	NR	Ref.	0.66		
7-11 cm	NR	1.11 (0.48-2.62)			
≥12 cm	NR	1.79 (0.51-6.27)			
CEA					
<5 (μg/L)	NR	Ref.	0.93		
≥5 (μg/L)	NR	1.04 (0.45-2.41)			
CA 19-9					
<34 (U/mL)	NR	Ref.	0.20		
≥34 (U/mL)	NR	2.09 (0.65-6.67)			
Surgical technique					
Anterior resection	NR	Ref.	0.07	Ref.	0.02
Abdominoperineal resection	NR	2.02 (0.92-4.43)		2.74 (1.13-6.66)	
Number of resected lymph nodes					
<12	NR	Ref.	0.68		
≥12	NR	0.85 (0.38-1.87)			
Tumor deposits					
+	NR	1.92 (0.77-4.83)	0.16		
-	NR	Ref.			
LVI					
+	NR	1.51 (0.68-3.37)	0.31		
-	NR	Ref.			
PNI					
+	NR	1.73 (0.72-4.14)	0.21		
-	NR	Ref.			
Surgical margin					
+	21.1 (5.6-36.5)	10.70 (3.43-33.37)	<0.001	12.94 (3.91-42.82)	<0.001
-	NR	Ref.		Ref.	
TRG					
Score 0+1	NR	0.91 (0.36-2.28)	0.84		
Score 2+3	NR	Ref.			
LIPI score					
Good	NR	Ref.	0.26		
Int/poor	NR	1.57 (0.71-3.47)			
Adjuvant CT					
+	NR	0.38 (0.16-0.91)	0.02	0.20 (0.07-0.55)	0.002
-	62.2 (25.4-98.9)	Ref.		Ref.	

## Discussion

This study analyzed the relationship between the LIPI, which indicates SIR, and treatment response, and also survival outcomes in LARC patients treated with NACRT. The results revealed that the int/poor LIPI score was significantly associated with good response and poor DFS. Additionally, APR and the presence of tumor deposit were independent poor risk factors for DFS, and APR, surgical margin positivity, and absence of adjuvant CT were for OS.

A well-known factor in the development and spread of tumors is the systemic inflammatory response, which can be reflected by inflammatory markers such as NLR, PLR, MLR, SII, and Glasgow prognostic score (GPS) [11,12]. Nevertheless, data on the relationship between inflammatory markers and treatment response are limited and inconsistent for solid tumors, including RC [12,20-22]. Studies mostly have analyzed the change in inflammatory markers before and after NACRT [11,22]. However, knowing the effect of basal values on treatment response may be more effective in determining the appropriate strategy for initial treatment. Dreyer et al. examined the association of pretreatment NLR, PLR, GPS, and neutrophil-to-platelet score (NPS) with treatment response in patients receiving NACRT for RC. They found that only increased GPS was associated with poor response [12]. Krauthamer et al. reported that low pretreatment NLR indicated good treatment response [20]. In the analysis of Shen et al., baseline NLR was not significantly associated with treatment response [21]. Lee et al. found that patients with an initially high PLR had a lower CR rate [22]. In the literature, there are significant differences in terms of radiation doses, CT regimens, clinical stage of patients, definition, and stratification of good responders, and cut-off values of relevant indexes. Thus, this heterogeneity leads to confusion in the comparison of the results. In the current study, the int/poor LIPI score, reflecting high inflammation, was associated with a good response.

There are several studies examining the relationship between inflammatory markers indicating SIR and survival outcomes in patients treated with NACRT for LARC. Most of these markers were found to be poor prognostic for DFS and OS [21,22]. LIPI and other mentioned inflammatory markers are calculated using biomarkers commonly used in daily clinical practice. Therefore, they are useful and easily accessible. However, almost all of the studies analyzing inflammatory markers are based on median values. The LIPI score is based on the ULN for LDH and "three" for dNLR as cut-off values. Therefore, it can be said that the cut-off value of LIPI is more standard than the mentioned markers that are based on the median values. In the present study, int/poor LIPI was associated with poor DFS relative to good LIPI, consistent with the literature. However, there was no significant relationship between LIPI and OS. Just as the event number for recurrence, the number of events for OS was limited in the study. Therefore, results may vary with longer follow-ups. The confusing point was that despite patients with int/poor LIPI scores had a higher good response rate, they had shorter DFS. Pathological good response to NACRT is expected to be associated with longer DFS for all study populations. However, a good response may not have predicted DFS in patients in the int/poor LIPI group with high inflammation.

The type of surgical procedure is another prognostic factor for RC. The majority of RCs present at a locally advanced stage and need extensive surgery. APR is more frequently performed than anterior resection (AR) for tumors located in the lower rectum and for tumors that have more locally advanced stages [23]. Therefore, APR is expected to be associated with shorter recurrence-free survival and OS compared to AR and this finding has been demonstrated by several studies [24]. In contrast, Omidvari et al. emphasized that AR and APR did not make a difference for DFS or OS in lower RC [25]. In the current study, we identified APR as an independent poor prognostic factor for both DFS and OS compared to AR.

Tumor deposits are described as extramural discontinuous cancer spreads without residual lymph node structures [26]. According to the 8th AJCC TNM staging system, tumors with regional lymph node-negative but tumor deposit-positive are defined as pN1c [27]. Tumor deposits are associated with prognosis as expected, and moreover, there are studies advocating that tumor deposits should be considered metastatic lymph nodes [28,29]. In this study, tumor deposits were associated with DFS consistent with the literature, while they were not associated with OS. The low number of events for OS may affect this result.

Adjuvant CT has been a widely investigated and debated issue before total neoadjuvant therapy became the standard treatment strategy. In the meta-analysis of individual patient data from four studies (i-CNR-RT, PROCTOR-SCRIPT, CHRONICLE, and EORTC 22921), including data from patients with (y)pTNM stage II or III disease, OS was similar between patients WHO received adjuvant CT and patients who underwent observation. And also adjuvant CT did not significantly improve DFS or distant recurrences [30]. However, in the mentioned meta-analysis, approximately 25% to 30% of patients did not receive adjuvant CT as intended. In the current study, OS was significantly shorter in patients who did not receive adjuvant CT compared to patients who received adjuvant CT.

Our study has significant limitations, primarily due to its retrospective nature and small sample size. Also, it was not possible to control all potential confounding biases. Due to the small sample size, we included the patients with intermediate and poor LIPI scores in the same group. In addition, analyses may have been affected as the numbers of events for DFS and OS were limited.

## Conclusions

In this study, we investigated the predictive and prognostic significance of LIPI in LARC patients treated with NACRT. The results indicated that an int/poor LIPI score was associated with good treatment response but shorter DFS. In other words, we confirmed the importance of LIPI in rectal cancer. The baseline LIPI score acts as an easily accessible and useful prognostic marker and it has a significant potential for making appropriate treatment decisions in LARC.
